# Silos and the Value of Ensilage1A summary of a practical talk at a meeting of the Keystone Veterinary Association, Philadelphia, February 9, 1897.

**Published:** 1897-06

**Authors:** T. D. Detrich

**Affiliations:** Flourtown, Pa.


### SILOS AND THE VALUE OF ENSILAGE.[1]

By Rev. T. D. Detrich,
FLOURTOWN, PA.

It is a sign of progress when theory and practice combine in order to arrive more nearly at the truths of nature. Science without experience seems to build castles in the air, and experience without science is a waste of time and great values. Happily the scientist, the producer, and the consumer are hand-in-hand to-day, working for one and the same end, and no one can say that their interests are any longer divided. It is for this reason that it is a pleasure for me as a layman to speak to you as professors in science, and in this way blend theory and practice, for we have reached a civilization that is no longer baptized with talk for talk's sake, but with talk for truth's sake.

The subject which your committee requested me to speak on was "Ensilage from a Practical Standpoint." In 1886 I wrote to Professor Miles, Massachusetts Experiment Station, asking for some reading-matter that would assist me in building a silo. He very kindly gave me the desired information, and I proceeded to make an air-tight wooden building. If I were to build one to-day, I would use the same material, but make it deep instead of long and narrow. The first corn ever put in was frosted, and I had as good ensilage as I ever fed. Major Alvord told me he had the same experience. For husking, the crop would have been ruined, but in the silo it was all

[1] A summary of a practical talk at a meeting of the Keystone Veterinary Association, Philadelphia, February 9, 1897.

saved. I can say that I was highly delighted when I opened the silo and found the ensilage excellent and the cattle eating it greedily. From that day to this I have looked upon corn-ensilage as a sure crop. The cause of the crop being caught by the frost was owing to the birds pulling it out three times.

The ground was heavily enriched, using all the manure made by two horses and seventeen head of cattle on two acres of ground from October 1st to May 1st. The corn was dropped three and four grains to the running foot in rows three feet apart. The variety was the large grained Southern hominy-corn, growing fourteen and sixteen feet high, one and two ears to the stalk, selecting this corn because of its great growth and having a sweet stalk, while the varieties of sweet corn have only a sweet ear. The crop is a perfect forest about the time it is ready for the silo. It is cut off down at the first joint with a hoe having a handle about eighteen inches long. The old-fashioned cutting-box machine with a narrow throat was soon laid aside for the sixty-inch throat ensilage-cutter that we saw at the World's Fair. Two men stand on the wagon and throw off grips of corn as rapidly at they can take it up, and the machine just devours it. This machine has practically solved the ensilage problem of cutting, while heretofore the cutting has always been a great bugbear to the siloist. Then, too, the machine does not chop the corn off in lengths of one-half or one-quarter inch, but it cuts, haggles, shreds, tears, and consequently distributes the grain all through the ensilage, and the carrier takes it into the silo.

In the early days of filling it was thought necessary to fill rapidly, tramp, and weight well. It is better to fill evenly and slowly, and if the corn is very dry to sprinkle with a watering-can as it goes up the carrier, and after the silo is full to wet the top with a few gallons twice a week, covering the ensilage with grass, chaff, or any thing that will cover a foot to fifteen inches and help exclude the air, for the ensilage will make its own cover. It is surprising how nicely the ensilage moulds a cover for itself. No bird can make a nest more neatly woven and not nearly so hermetically sealed from the air as ensilage makes its own cover. Every honest man will admit that the top for several inches spoils, and some fifteen to eighteen inches in the corners.

Now the opening of a silo is a more particular job than the putting in of the fodder. When a silo is opened all the bad black stuff in the corners, sides, and top should be taken off and out at once. No one should go into a silo and commence to dig as though he were digging garden in the centre of a bed, but he must scrape and fork off lightly the whole top, corners, and sides till all the bad stuff is out. Then let him take off the woven covering as it appears, and underneath is the perfectly kept ensilage, emitting a smell like a sugar-house on Delaware Avenue; and the whole silage should be taken off of the top to the depth of two or three inches a day after the silo is well opened; it will keep, and feed sweet succulent June pasture till it is empty.

We always feed it three times a day in a balanced ration of hay, bean gluten meal, linseed meal, and decorticated cottonseed meal, and regard it a most wholesome food for the Jerseys. The flavor of the milk and condition of the animals' health are all that can be desired. They relish ensilage above all other food; occasionally we feed all ensilage and no hay. Then to test the cow's fondness for the ensilage we take clean, ripe Baldwin apples and cut them up, and in every instance the ravenous fawn Jersey would smell over the apples in one end of the trough, but leave the apples and turn to the ensilage. There is evidently a difference between a chemist's crucible and a cow's stomach. We see this in the different experiment stations, estimating the value of dry fodder, corn, and ensilage to the same; but the cow knows the difference, and it seems to be the same kind of a preference that we have for a fresh June strawberry over the one that is dried or evaporated and water poured over when offered for food.

As for the cows, we do not know that we have any record-breakers, but are sure we have profit-makers; and ensilage keeps up the flow of milk, because it is nearest to pasture. It is better than dry fodder, because it is more digestible, and undoubtedly takes the place of roots.

It is the most economical way to handle the corn crop.

1. It is the least waste—for the stalks, ear, leaf, and tassel are all saved. There is no fodder-rick to build, no grist to grind, no corn to husk, no soft corn and no hard corn; no long stalks in the manure, and no bundles to tie.

2. The smallest space. A ton of cornstalks takes up 900 cubic feet of room; a ton of hay 500, and a ton of ensilage 45 cubic feet. Two tons of hay to the acre are called a good crop, while it is easy to raise twenty-five to thirty tons of corn-ensilage on the same ground the way we manure.

3. It is the cheapest base for a ration just for the reasons stated. It is no longer thought to be fancy farming to have a silo, but an indispensable necessity for the successful and profitable keeping of the dairy cow.

[The lecturer then passed around through the audience two jars of ensilage, representing the two methods of cutting, the shredded fodder emitting much the sweeter odor, which was accounted for by the close packing of the crushed stalks, ears, and fodder over the method of cutting in cubes, for both specimens were cut at the same time.]

